# No better moment to score a goal than just before half time? A soccer myth statistically tested

**DOI:** 10.1371/journal.pone.0194255

**Published:** 2018-03-08

**Authors:** Stijn Baert, Simon Amez

**Affiliations:** 1 Ghent University, Ghent, Belgium; 2 Research Foundation–Flanders, Brussels, Belgium; 3 University of Antwerp, Antwerp, Belgium; 4 Université Catholique de Louvain, Louvain-la-neuve, Belgium; 5 IZA, Bonn, Germany; 6 GLO, Maastricht, Netherlands; 7 IMISCOE, Rotterdam, Netherlands; University of Muenster, GERMANY

## Abstract

We test the soccer myth suggesting that a particularly good moment to score a goal is just before half time. To this end, rich data on 1,179 games played in the UEFA Champions League and UEFA Europa League are analysed. In contrast to the myth, we find that, conditional on the goal difference and other game characteristics at half time, the final goal difference at the advantage of the home team is 0.520 goals lower in case of a goal just before half time by this team. We show that this finding relates to this team’s lower probability of scoring a goal during the second half.

## Introduction

Soccer is big business, especially in Europe. In 2015, the English Premier League—the primary soccer competition in the United Kingdom—sold the television rights to its games for the period 2016–2019 for 1.712 billion pounds per year, or 10.190 million pounds per game to be shown live [1]. Depending on the final league position and the number of games shown live, both driven by team achievement, the share for each team in the Premier League varies from about 50 to about 100 million pounds per year [2]. So, the difference between winning and losing a soccer game may amount, economically speaking, to a lot of money.

Therefore not surprisingly, during the past two decades, scholars have shown a growing interest in the determinants of soccer game results such as goal difference and winning probability. In this context, sports economists, sports psychologists, and sports scientists have linked soccer achievements to (i) team and coach ability; (ii) team effort; (iii) the mental and physical condition of the team players; and (iv) choices made by these players, their coach and the referee [3–7]. Besides their direct impact on achievements, these factors potentially mediate the impact of the following team and game characteristics: (a) relative salaries of the players [8–9]; (b) age and race composition of the competing teams [10]; (c) playing as home or away team [11–17]; (d) bias in referee decisions [17–25]; (e) geographical distance between home and away team municipality [26–27] (f) wearing red shirts [28–30]; (g) in-season head coach dismissals [31–37]; and (h) culturally driven game tactics [38]. On the one hand, this literature confirms pearls of soccer “wisdom” such as the idea that German teams often score in the last minute(s) of a game [38] and that referees tend to favour home teams [39–42]. On the other hand, no robust evidence is found for the old soccer myth that team performance is positively affected by the expulsion of a player (after obtaining a red card) [43–45].

However, surprisingly, one of most well-established soccer myths—namely that a particularly good moment to score a goal is just before half time—is yet to be exposed to an empirical test. This is the aim of the present study. Soccer reporters often link scoring just before half time to a “self-esteem boost” and a “psychological blow” with which the scoring team and the team that conceded a goal, respectively, are sent to the locker rooms. If this event does, indeed, compared with scoring and/or conceding a goal earlier, have a distinctive impact on perceived pressure and self-consciousness, it may affect performance during the second half [16,46–49]. In addition, the emotional treatment of a goal just before the break may also (positively or negatively) affect the tactical choices made during the break and further second half preparations [50].

Two studies have indirectly investigated the empirical value of this soccer myth. Firstly, Ayton and Braennberg [46] investigated whether the final outcome of 355 English premier leagues games, which were poised 1–0 at half time, depended on when the first goal was scored. Secondly, Heuer and Rubner [51] studied the general effect of the timing of (and time passed since) previous goals on the goal rate in soccer games in the German Bundesliga. None of both found significant evidence for the absolute times of previous goals as a determinant of later game outcomes.

In this study we directly test the aforementioned soccer myth. More concretely, we answer the following research question: “Conditional on the score at half time and other game and team characteristics, does scoring a goal just before half time affect final game outcomes?” To tackle this question, we employ an international and unique data set containing all games played in the UEFA Champions League between 2008 and 2014 and all games played in the UEFA Europa League between 2011 and 2014. These data are analysed by means of linear (probability) regressions of final game outcomes on the event of a goal just before half time, other game events and characteristics, and country or team fixed effects. Various definitions of scoring “just before half time” are used and the effects of this scoring behaviour on various final game outcomes are compared. In addition, our statistical framework allows us to measure heterogeneous effects by other game events and characteristics. Finally, we also test the alternative soccer myth that just after the break is a particularly good moment to score a goal.

## Methods

### 2.1 Data

Our data set was constructed by merging two data sources publicly available from the official website of the Union of European Football Associations (UEFA; http://www.uefa.com). Our first source of data was reports of games in the UEFA Champions League and the UEFA Europa League. Both are European soccer competitions organised by UEFA and contested by top-division European teams. The number of teams from each nation that enters into (the qualification rounds of) these competitions is based on the performance of the country’s teams in former editions of both competitions. The teams that finished in the highest position(s) in the former season of each nation’s top level league (and, for some nations, their cup competition) are eligible for the (qualification rounds of) the UEFA Champions League; those who finish next in line are eligible for the UEFA Europa League. The UEFA Champions League and UEFA Europa League begin with a group stage of 32 and 48 teams, respectively, divided into groups of four teams. Each team meets the others in its group at home and away. The group winners and runners-up of each group proceed to the knock-out phase of their competition that culminates with a final game. In addition, the third-placed team of each UEFA Champions League group enters the UEFA Europa League knock-out phase. The group stage of each season is played from September to December, the knock-out stage from February to May. For more information on the modalities of the two competitions and the rules of a soccer game, we refer to the official UEFA website (http://www.uefa.com) and to FIFA [52].

During one soccer season, in total 125 and 205 official games are played in the group phase or knock-out phase of the UEFA Champions League and UEFA Europa League, respectively. Combining information for the UEFA Champions League between 2008 and 2014 and the UEFA Europa League between 2011 and 2014—for both competitions we used all data for which reports were available at the start of our research—yielded reports on 1,365 games between 148 teams. We excluded 186 games without a substantial competitive value. We defined games without a substantial competitive value as games in the group phase if, for one of the teams, it was mathematically impossible to change its qualification status for the next round. This is the case if a team is sure it will finish the group stage (i) as winner or runner-up of its group in the UEFA Champions League or UEFA Europa League; (ii) third placed in its group in the UEFA Champions League; (iii) fourth placed in its group in the UEFA Champions League; or (iv) third placed or fourth placed in its group in the UEFA Europa League. We come back to the sensitivity of our statistical analyses by this restriction to games with a substantial competitive value in Section 3.4.

To answer our research question, various variables capturing the game outcomes at full time and indicators of whether a goal was scored just before half time were derived from the aforementioned game reports. In addition, various control variables, potentially correlated with both game characteristics, were constructed. Table 1 presents descriptive statistics for the variables used in the regression analyses below. The variables used as dependent variables are presented in Panel A, our main explanatory variables in Panel B and the control variables in Panel C.

**Table 1:** Data: Summary statistics.

	Mean	Standard deviation
***A*. *Dependent variables***		
Goal difference at full time	0.386	1.835
Victory by home team	0.457	0.498
Victory by away team	0.293	0.456
Final number of goals by home team	1.527	1.299
Final number of goals by away team	1.141	1.136
First goal in second half by home team	0.455	0.498
First goal in second half by away team	0.330	0.470
***B*. *Independent variables*: *Main explanatory variables***		
Goal home team between start of minute 45 and half time^a^	0.041	0.198
Goal away team between start of minute 45 and half time	0.036	0.188
Goal home team between start of minute 44 and half time	0.051	0.220
Goal away team between start of minute 44 and half time	0.047	0.211
Goal home team between start of minute 43 and half time	0.066	0.249
Goal away team between start of minute 43 and half time	0.055	0.228
Goal home team between start of minute 42 and half time	0.079	0.270
Goal away team between start of minute 42 and half time	0.066	0.249
Goal home team between start of minute 41 and half time	0.091	0.287
Goal away team between start of minute 41 and half time	0.076	0.266
First goal in second half by home team before minute 51	0.064	0.246
First goal in second half by away team before minute 51	0.040	0.196
***C*. *Independent variables*: *Other game characteristics***		
Goal difference at half time	0.187	1.140
Number of goals home team during first half	0.680	0.847
Last goal in first half by home team	0.390	0.488
Last goal in first half by away team	0.282	0.450
Game in UEFA Europa League	0.444	0.497
Game in group phase	0.697	0.460
Red card in first half for home team	0.013	0.112
Red card in first half for away team	0.032	0.177
Goal difference at start of minute 51	0.212	1.196
Number of goals home team before minute 51	0.746	0.882
Last goal before minute 51 by home team	0.393	0.489
Last goal before minute 51 by away team	0.279	0.449
Red card before minute 51 for home team	0.019	0.135
Red card before minute 51 for away team	0.038	0.192
Relative strength home team	0.004	1.722
N	1,179	

Notes: A definition of these variables can be found in Section 2.1.

^a^As mentioned in Section 2.1, if both the home and away team score during this time window, only the team that scores the last goal gets a 1-value for the indicator of scoring just before half time.

In line with, for example, Balduck et al. [31], Koning [34], Mechtel et al. [44] and van Ours and van Tuijl [37], we use the goal difference at full time as the dependent variable for our benchmark statistical model. This variable is defined as the final difference between the goals scored by the home team and the goals scored by the away team. Its average value of 0.386 confirms the aforementioned home advantage found in the literature. The summary statistics for our alternative dependent variables go in the same direction: the probability of victory (45.7% versus 29.3%), the final number of goals (1.527 versus 1.141) and the probability of being the first team to score a goal in the second half (0.455 versus 0.330) are higher for the home team.

In our benchmark regression model, we define scoring a goal just before half time as scoring a goal between the start of minute 45 and the end of the first half. This time window lasts at least one minute but usually some extra time is added at the discretion of the referee. This extra time varies from a few seconds to a few minutes and compensates for game interruptions due to, for example, substitution of players, stoppage of play because of a serious injury, and time-wasting by a team [52]. By our definition, we align ourselves with van Ours and van Tuijl [38], who defined the “dying seconds” of a game as the time between the start of minute 45 of the second half and full time.

However, in Section 3.2, we will compare the effect of scoring a goal just before half time following this definition on full time outcomes, keeping other game characteristics constant, with the corresponding effect of scoring a goal just before half time following alternative definitions. More concretely, for our alternative definitions, “just before half time” begins at the start of minute 44, 43, 42 or 41, instead of at the start of minute 45. If both the home and away team score during this time window (which occurs in five—0.4%—of the games in our sample for the broadest definition of “just before half time”), only the team that scores the last goal gets a 1-value for the indicator of scoring just before half time. Depending on the definition used, in 4.1% to 9.1% of the games in our sample, the home team scores just before half time, while in 3.6% to 7.6% of the cases, the away team scores just before half time. In the most strict (broad) definition, there is a goal just before half time in 91 (197) “treated” observations while there are 1,088 (982) control games. So, the broader definitions yield more variation in the treatment indicator (and ipso facto more statistical power for our analyses) at the cost of being farther away from what is, intuitively, “just before half time” in the strict sense.

Finally, to test the alternative soccer myth that just after the break is a particularly good moment to score a goal, we also constructed indicators for scoring a first goal in the second half during the first five minutes of this second half as a home team or as an away team. In 10.4% of the games, at least one goal is scored during this period—in 6.4% of the games by the home team, and in 4.0% of the games by the away team.

In all our statistical analyses, we condition the effect of scoring just before half time on the score at half time. This is done by controlling for the goal difference at half time and the number of goals scored by the home team at that moment. As a consequence, we compare the effect of scoring a last goal (that is, without the other team scoring a later goal during the first half) just before half time on full time outcomes with the effect of scoring a goal at a different moment during the first half.

In addition, from more extended versions of our regression model onwards, we control for indicators of scoring a last goal at any time during the first half by the home or away team. When not controlling for these variables, our treatment effect—that is, the effect of scoring just before half time on full time outcomes—could just reflect a general (dis)advantage of being the team which scores the last goal of the first half given the score at half time. As a consequence, when these control variables are included, we compare the effect of scoring a (last) goal just before half time on full time outcomes with the effect of scoring a last goal of the first half at a different moment during this first half. Moreover, we include a variable indicating whether the game is a game in the UEFA Europa League and a variable indicating whether the game is a game in the group phase of one of the two competitions. Finally, two variables indicating whether the home and away team received a red card during the first half are included.

With respect to the investigation of the alternative soccer myth, we condition on the mentioned intermediate outcomes (goal difference, number of goals of the home team, a last goal in the first half by the home team, a last goal in the first half by the away team, a red card for the home team and a red card for the away team) at the start of minute 51—that is, immediately after the first five minutes of the second half.

To be able to control for the relative strength of the home and away teams (and to investigate heterogeneous effects of scoring just before half time by relative team strength), we merged the presented game information with information on the relative UEFA team coefficient of both teams. The UEFA coefficient of a team is based on its participation and results in the five previous seasons of the UEFA Champions League and UEFA Europa League. Our proxy of relative strength of the home team of a game is the natural logarithm of the quotient of the home and away teams’ UEFA team coefficient for that season plus 1 (to avoid division by 0 for teams who did not participate in one of the two European competitions during the five previous seasons). In the next subsection, we present additional ways to control for team heterogeneity. Furthermore, in Section 3.4, we elaborate on various robustness analyses conducted in which alternative control variables were used.

The data is available as S1 Dataset.

### 2.2 Econometric model

To answer our research question, the data presented in the former subsection are analysed by linear regression models which can be abstracted by means of the following general equation:

$$Y_{ijtn}=\alpha +\beta X_{ijtn}+\delta D_{ijtn}+\mu_{i}+\nu_{j}+\varepsilon_{ijtn}.$$ (1)

In this equation, *Y*_*ijtn*_ represents the dependent variable: a (final) outcome of the nth game between home team i and away team j in season t. ***D***_***ijtn***_ is a vector of two independent variables of which we want to know the effect with respect to *Y*_*ijtn*_. More concretely, this is a set of two variables indicating whether the home and away team score a goal just before half time. ***X***_***ijtn***_ is a vector of variables that may also affect the final game result and that may be correlated with ***D***_***ijtn***_. By including the relevant variables from Panel C of Table 1, we control for the endogeneity of scoring just before half time with respect to the final game outcomes. ***X***_***ijtn***_ contains both a variable which is constant across games played during a particular season (that is, the relative strength of the home and away teams) and variables which are game-specific. *α* is the intercept of the model and *ε*_*ijtn*_ is the error term.

Finally, *μ*_*i*_ (*ν*_*j*_) is a home (away) team fixed effect. By means of introducing these fixed effects, we essentially estimate the effect of scoring just before half time on full time achievements within teams. As a consequence, all dimensions of unobserved, time-constant team heterogeneity that may determine full time outcomes and that may correlate with scoring behaviour just before half time are controlled. However, the effect of scoring just before half time is in these fixed effects models exclusively identified based on the information for (home and away) teams for which we observe at least one game in which this team or its competitor scores a goal just before half time and one game in which this is not the case. This results in a lower level of statistical power. Therefore, in intermediate versions of our statistical model, we control (related to the relative strength of the national competitions and the culturally driven game tactics mentioned in Section 1) for fixed effects at the country of the home and away team level only.

For each of the models presented in Section 3, we computed multicollinearity diagnostics leading to variance inflation factors lower than 10. In case a binary dependent variable was used, standard errors were White-corrected. In Section 3.4, we elaborate on the robustness of our findings based on regression model (1) to alternative (non-linear) model specifications.

## Results

### 3.1 Benchmark model

Table 2 presents the results of regressing the goal difference at full time on indicators of scoring behaviour just before half time (following our strict sense definition) and various sets of control variables. Based on the soccer myth predicting a positive effect of scoring just before half time, a significantly positive (negative) effect of a goal just before half time by the home (away) team on the goal difference at full time is expected.

**Table 2:** Results: Benchmark model.

	(1)	(2)	(3)	(4)	(5)
**Goal home team between start of minute 45 and half time**	-0.330* **(0.195)**	-0.362* **(0.199)**	-0.351* **(0.195)**	-0.396** **(0.195)**	-0.520** **(0.210)**
**Goal away team between start of minute 45 and half time**	**-0.082 (0.206)**	**-0.014 (0.213)**	**-0.042 (0.209)**	**-0.011 (0.209)**	**0.102 (0.229)**
Goal difference at half time	1.123*** (0.052)	1.065*** (0.081)	0.992*** (0.080)	0.943*** (0.081)	0.955*** (0.091)
Number of goals home team during first half	0.045 (0.071)	0.058 (0.106)	0.092 (0.104)	0.065 (0.104)	-0.007 (0.115)
Last goal in first half by home team		0.093 (0.133)	0.098 (0.130)	0.098 (0.130)	0.222 (0.142)
Last goal in first half by away team		-0.099 (0.154)	-0.131 (0.151)	-0.043 (0.150)	0.016 (0.167)
Game in UEFA Europa League		0.062 (0.077)	0.054 (0.075)	0.021 (0.086)	-0.041 (0.147)
Game in group phase		-0.042 (0.083)	-0.049 (0.081)	-0.060 (0.088)	-0.044 (0.102)
Red card in first half for home team		-0.493 (0.337)	-0.543 (0.330)	-0.670** (0.325)	-0.883** (0.362)
Red card in first half for away team		0.095 (0.214)	0.161 (0.210)	0.132 (0.210)	-0.034 (0.233)
Relative strength home team			0.155*** (0.022)	0.109*** (0.024)	-0.024 (0.093)
Intercept	0.161*** (0.055)	0.159 (0.097)	0.171 (0.155)	0.231 (0.163)	0.453 (0.351)
Fixed effects country home team	No	No	No	Yes	No
Fixed effects country away team	No	No	No	Yes	No
Fixed effects home team	No	No	No	No	Yes
Fixed effects away team	No	No	No	No	Yes
Dependent variable: Goal difference at full time	Yes	Yes	Yes	Yes	Yes
R^2^	0.505	0.508	0.528	0.582	0.671
N	1,179	1,179	1,179	1,179	1,179

Notes: The presented statistics are linear regression model estimates. The estimation results for the model’s main explanatory variables are in bold. A definition of the variables adopted in the regressions can be found in Section 2.1. Standard errors are between parentheses.

*** (**) ((*)) indicate significance at the 1% (5%) ((10%)) significance level.

In regression (1), we only control for the score at half time (goal difference and number of goals by the home team at half time). From model (2) onwards, the additional controls for other game events and characteristics mentioned in Section 2.1 are added. From model (3) on, our proxy of relative strength of the home team is also included. Finally, model (4) and model (5) control additionally for fixed effects at the country and team level, respectively.

The estimation results with respect to our main explanatory variables are robust across the five regression models. On the one hand, and in contrast to the related soccer myth, a significantly negative effect of scoring a goal just before half time by the home team is found with respect to the goal difference at full time. This effect is higher in magnitude and more significant when country or team fixed effects are added. For our most extensive model, we find that a goal just before half time by the home team lowers the goal difference by 0.520 goals, ceteris paribus. With respect to the effect of a goal just before half time by the away team, we do not find a statistically significant effect on the goal difference at full time.

One might wonder whether the insignificant coefficient for a goal just before half time by the away team should be interpreted as a true null effect or as a signal of a lack of statistical power. Indeed, it is unpredictable whether significant coefficients for this variable would emerge in case our number of observations would be larger. However, the coefficient for a goal just before half time by the away team is also in economic terms insignificant. More concretely, it varies between -0.082 (model (1)) and 0.102 (model (5)) across the models presented in Table 2. So, at most, a treatment effect of a tenth of a goal is measured. Moreover, across models (1) to (5), standard errors of about 0.200 are measured for the two coefficients related to scoring a goal just before half time by the home and away team. This means that effects with an absolute value of about 0.392 = 0.200*1.960; the latter factor approximates the 97.5 percentile point of the normal distribution) or more could be rejected at the 5% significance level. We believe this is fair–at least we have enough statistical power to reject the null hypothesis of a null treatment effect with respect to scoring a goal just before half time by the home team. We elaborate on potential explanations for the treatment effects measured in Section 4.

Before inspecting the sensitivity of the mentioned main findings to alternative variables capturing achievements at full time and scoring behaviour just before half time, we briefly discuss some secondary results reported in Table 2. Firstly, a highly significant association of goal difference at full time with the goal difference at half time, with the expected positive sign, is found. Conditional on goal difference at half time, the number of goals scored by the home team (and ipso facto the number of goals by the away team) at half time has no effect on the goal difference at full time. Secondly, there is a negative effect (significant when controlling for country or team fixed effects) of a red card for the home team, whilst no effect of a red card for the away team is found. This finding is remarkably consistent with Mechtel et al. [44], who found that the expulsion of one of their players had a negative impact on the home team’s performance but a mixed impact, depending on the time remaining after the sending-off, for the away team. Thirdly, the effect of the (time-varying) relative strength of the home team becomes statistically insignificant when (time-constant) fixed effects at the home and away team level are included.

### 3.2 Alternative dependent and independent variables

In this subsection, we test the sensitivity of our main finding of a negative (neutral) effect of scoring just before half time by the home (away) team on full time achievement to alternative proxies of our main explanatory and dependent variables. In addition, the alternative soccer myth of a premium of scoring immediately at the start of the second half is exposed to an empirical test. Throughout this subsection, regression model (5) of Table 2 is used as our benchmark regression.

Table 3 presents our regression results when adopting the less strict definitions of “just before half time” mentioned in Section 2.1. Our main finding presented in Section 3.1 turns out to be robust to the various definitions used. The result of regressing goal difference at full time on scoring behaviour between the start of minute 42 (instead of minute 45) and half time is an exception, as the effect of a goal during this time window by the home team becomes insignificant. However, with a p-value of 0.102, this coefficient also tends towards significance, albeit only at the 10% significance level.

**Table 3:** Results: Alternative main explanatory variables.

	(1)	(2)	(3)	(4)
**Goal home team between start of minute 44 and half time**	-0.493*** (0.192)			
**Goal away team between start of minute 44 and half time**	-0.043 (0.208)			
**Goal home team between start of minute 43 and half time**		-0.322* (0.172)		
**Goal away team between start of minute 43 and half time**		0.145 (0.195)		
**Goal home team between start of minute 42 and half time**			-0.261 (0.159)	
**Goal away team between start of minute 42 and half time**			0.058 (0.182)	
**Goal home team between start of minute 41 and half time**				-0.341** (0.154)
**Goal away team between start of minute 41 and half time**				0.085 (0.175)
Goal difference at half time	0.961*** (0.091)	0.961*** (0.091)	0.967*** (0.091)	0.963*** (0.091)
Number of goals home team during first half	-0.008 (0.115)	-0.014 (0.117)	-0.012 (0.117)	-0.007 (0.117)
Last goal in first half by home team	0.227 (0.143)	0.225 (0.143)	0.212 (0.143)	0.237 (0.144)
Last goal in first half by away team	0.046 (0.167)	0.012 (0.167)	0.032 (0.168)	0.018 (0.168)
Game in UEFA Europa League	-0.036 (0.147)	-0.040 (0.147)	-0.035 (0.147)	-0.032 (0.147)
Game in group phase	-0.045 (0.102)	-0.050 (0.102)	-0.050 (0.102)	-0.049 (0.102)
Red card in first half for home team	-0.871** (0.362)	-0.901** (0.362)	-0.893** (0.362)	-0.896** (0.362)
Red card in first half for away team	-0.042 (0.233)	-0.032 (0.233)	-0.032 (0.234)	-0.039 (0.233)
Relative strength home team	-0.022 (0.093)	-0.022 (0.093)	-0.022 (0.093)	-0.022 (0.093)
Intercept	0.452 (0.351)	0.453 (0.352)	0.466 (0.352)	0.455 (0.351)
Fixed effects home team	Yes	Yes	Yes	Yes
Fixed effects away team	Yes	Yes	Yes	Yes
Dependent variable: Goal difference at full time	Yes	Yes	Yes	Yes
R^2^	0.671	0.670	0.669	0.670
N	1,179	1,179	1,179	1,179

Notes: The presented statistics are linear (probability) regression model estimates. The estimation results for the model’s main explanatory variables are in bold. A definition of the variables adopted in the regressions can be found in Section 2.1. Standard errors are between parentheses.

*** (**) ((*)) indicate significance at the 1% (5%) ((10%)) significance level.

On the other hand, when regressing the goal difference at full time on indicators of scoring behaviour by the home and away teams between the start of minute 44 (instead of minute 45) and half time, the effect of a home goal during this time window, keeping the most extensive set of controls constant, is even more significant than the one found for our benchmark regression. This is related to a decrease in the standard error for this variable (and thereby to the higher variation in this variable, as mentioned in Section 2.1) compared to the corresponding variable in our benchmark regression and not to an increase in the absolute value of the estimated coefficient (which also slightly decreases, that is, from 0.520 to 0.493).

With respect to the effect of a goal by the away team, a non-significantly positive effect on the goal difference at full time is found for four of the five (benchmark and alternative) definitions of “just before half time”. This is, to some extent, consistent with the empirical pattern mentioned in the previous paragraphs, as a higher goal difference (that is, as mentioned in Section 2.1, the number of goals scored by the home team minus the number of goals scored by the away team) at full time is to the disadvantage of the away team.

Next, in the first two columns of Table 4, we present the results of regressing victory by the home team (column (1)) and victory for the away team (column (2)) on the set of variables included in our benchmark regression. In line with its effect on the goal difference at full time, a goal just before half time by the home team lowers the chances of a victory for this team by 8.7 percentage points and increases the chances of a victory for the away team (and ipso facto a loss for the home team) by 10.7 percentage points, ceteris paribus. However, only the latter effect is statistically significant and this at the 10% significance level only. In addition, and in line with the results presented in Table 2, no evidence is found for an effect of a goal just before half time by the away team. So, again, no evidence is found overall for the soccer myth investigated in this study, but the opposite dynamic is less outspoken with respect to victory chances than with respect to goal difference at full time.

**Table 4:** Results: Alternative dependent variables.

	(1)	(2)	(3)	(4)	(5)	(6)
**Goal home team between start of minute 45 and half time**	**-0.087 (0.068)**	0.107* **(0.063)**	-0.537*** **(0.152)**	**-0.017 (0.138)**	-0.232*** **(0.083)**	**0.121 (0.079)**
**Goal away team between start of minute 45 and half time**	**0.088 (0.081)**	**-0.043 (0.076)**	**0.154 (0.166)**	**0.051 (0.150)**	**0.124 (0.091)**	**-0.047 (0.086)**
Goal difference at half time	0.153*** (0.027)	-0.148*** (0.027)	-0.051 (0.066)	-1.006*** (0.060)	-0.052 (0.036)	-0.002 (0.034)
Number of goals home team during first half	0.018 (0.034)	0.043 (0.034)	1.010*** (0.083)	1.017*** (0.076)	0.009 (0.046)	0.017 (0.043)
Last goal in first half by home team	0.136*** (0.049)	-0.066 (0.043)	0.159 (0.103)	-0.063 (0.093)	0.085 (0.056)	-0.057 (0.053)
Last goal in first half by away team	-0.015 (0.053)	0.117** (0.057)	-0.023 (0.121)	-0.040 (0.109)	-0.026 (0.066)	-0.010 (0.062)
Game in UEFA Europa League	0.008 (0.051)	0.030 (0.047)	0.015 (0.107)	0.056 (0.097)	0.053 (0.058)	0.007 (0.055)
Game in group phase	-0.020 (0.034)	-0.025 (0.032)	-0.025 (0.074)	0.019 (0.067)	0.057 (0.040)	-0.048 (0.038)
Red card in first half for home team	-0.139 (0.124)	0.122 (0.126)	-0.505* (0.262)	0.038 (0.237)	-0.246* (0.143)	0.271** (0.136)
Red card in first half for away team	-0.169** (0.081)	0.025 (0.70)	-0.125 (0.169)	-0.091 (0.153)	0.089 (0.092)	-0.032 (0.087)
Relative strength home team	-0.006 (0.032)	0.008 (0.028)	-0.007 (0.067)	0.016 (0.061)	0.023 (0.037)	0.023 (0.035)
Intercept	0.496*** (0.126)	0.326** (0.131)	1.394*** (0.254)	0.940*** (0.230)	0.503*** (0.139)	0.377*** (0.132)
Fixed effects home team	Yes	Yes	Yes	Yes	Yes	Yes
Fixed effects away team	Yes	Yes	Yes	Yes	Yes	Yes
Dependent variable: Victory by home team	Yes	No	No	No	No	No
Dependent variable: Victory by away team	No	Yes	No	No	No	No
Dependent variable: Final number of goals by home team	No	No	Yes	No	No	No
Dependent variable: Final number of goals by away team	No	No	No	Yes	No	No
Dependent variable: First goal in second half by home team	No	No	No	No	Yes	No
Dependent variable: First goal in second half by away team	No	No	No	No	No	Yes
R^2^	0.528	0.499	0.656	0.631	0.302	0.296
N	1,179	1,179	1,179	1,179	1,179	1,179

Notes: The presented statistics are linear (probability) regression model estimates. The estimation results for the model’s main explanatory variables are in bold. A definition of the variables adopted in the regressions can be found in Section 2.1. Standard errors, White-corrected in case of binary outcomes variables, are between parentheses.

*** (**) ((*)) indicate significance at the 1% (5%) ((10%)) significance level.

Furthermore, in model (3) (model (4)) the final number of goals by the home (away) team is used as a dependent variable; in model (5) (model (6)) the probability of a first goal in the second half by the home (away) team is used as a dependent variable. We find that a goal just before half time by the home team, keeping the score at half time and other controls constant, lowers the final number of goals by this team by 0.537 and its probability of scoring in the second half before a goal by the away team is conceded by 23.2 percentage points. These effects are in line with our benchmark results and highly significant. In addition, no effect of scoring behaviour just before half time is found with respect to the goals scored by the away team during the second half. As a consequence, the findings presented in the latter columns of Table 4 give an insight into the dynamics underlying our main finding: home teams who score just before half time end up with a less beneficial goal difference due to their lower probability of scoring a goal during the second half (rather than due to a higher probability of scoring a goal during the second half by the away team).

In addition, for model (1) of Table 4, we find a negative effect of a red card for the away team during the first half on the final victory probability of the home team. So, an away team that gets a red card is, all other game events and characteristics kept constant, better off (that is, their probability of losing the game is lower). This result also to some extent corroborates the aforementioned findings of Mechtel et al. [44]. Moreover, in line with the discussion of our secondary results reported in Table 2, we find a negative effect of a red card for the home team on its final number of goals and its probability of scoring the first goal in the second half.

To test the alternative soccer myth (that a particularly good moment to score a goal is immediately at the start of the second half), we regress, by analogy with our benchmark regression, the goal difference at full time on variables indicating a goal by the home (away) team during the first five minutes of the second half. We return to alternative operationalisations of this alternative myth in Section 3.4. In addition, we control for the game events and characteristics as they are observed at the end of this time window—that is, at the start of minute 51 (minute six of the second half). However, as can be seen in Table 5, also with respect to this second soccer myth, we do not find any supporting evidence based on our sample of 1,179 recent games in the UEFA Champions League and UEFA Europa League. The scoring behaviour of neither of the two competing teams has a significant effect on the goal difference at full time. Given that neither myth survives the confrontation with our statistical analyses, one could wonder whether another particular time window during the first half is the best in which to score a goal. Therefore, we run additional regressions in which nine (three) indicators of goal scoring behaviour during subsequent time windows of five (15) minutes were introduced. However, conditional on the score at half time, no time window turned out to be statistically significantly more appropriate to score a goal than any other.

**Table 5:** Results: Alternative soccer myth.

	(1)
**First goal in second half by home team before minute 51**	**-0.015 (0.178)**
**First goal in second half by away team before minute 51**	**0.235 (0.227)**
Goal difference at start of minute 51	0.984*** (0.087)
Number of goals home team before minute 51	-0.000 (0.111)
Last goal before minute 51 by home team	0.089 (0.137)
Last goal before minute 51 by away team	0.053 (0.159)
Game in UEFA Europa League	0.005 (0.144)
Game in group phase	-0.055 (0.099)
Red card before minute 51 for home team	-0.674** (0.288)
Red card before minute 51 for away team	0.182 (0.209)
Relative strength home team	-0.030 (0.091)
Intercept	0.340 (0.344)
Fixed effects home team	Yes
Fixed effects away team	Yes
Dependent variable: Goal difference at full time	Yes
R^2^	0.687
N	1,179

Notes: The presented statistics are linear regression model estimates. The estimation results for the model’s main explanatory variables are in bold. A definition of the variables adopted in the regressions can be found in Section 2.1. Standard errors are between parentheses.

*** (**) ((*)) indicate significance at the 1% (5%) ((10%)) significance level.

### 3.3 Heterogeneous effects

In this subsection, we explore dimensions of potential heterogeneity in our main finding of a negative effect of a goal just before half time by the home team on the final goal difference, keeping the score at half time and other game events and characteristics constant. Firstly, in model (1) of Table 6, we interact the variable indicating a goal just before half time by the home team with our variable capturing the relative strength of this home team. We do this as, intuitively, one could expect that if the home team is a lot stronger, the timing of goals will not make a substantial difference with respect to the final game outcomes. Related, in model (2), we interact our treatment indicator with the goal difference at half time. Again, the idea is that if the goal difference is very high (to the advantage of the home team), the emotion brought about by a goal just before half time will not transcend the overall emotion of the high goal difference at half time. Finally, in model (3), we interact our treatment indicator with a variable indicating that the goal scored just before half time led to a transition from a tie to a leading position. In that case, the (psychological) treatment (and its effect) is expected to be higher in magnitude. For reasons of comparability of the regression results in Table 6, all explanatory variables that are interacted with “Goal home team between start of minute 45 and half time” are normalised by subtracting their average among the games for which our treatment indicator is 1. However, none of the interactions presented in Table 6 turns out to be statistically significantly different from 0.

**Table 6:** Results: Heterogeneous effects.

	(1)	(2)	(3)
**Goal home team between start of minute 45 and half time**	-0.530** **(0.210)**	-0.517** **(0.210)**	-0.516** **(0.210)**
**Goal home team between start of minute 45 and half time x Relative strength home team (normalised)**	**-0.026 (0.024)**		
**Goal home team between start of minute 45 and half time x Goal difference at half time (normalised)**		**-0.230 (0.239)**	
**Goal home team between start of minute 45 and half time x Positive goal difference thanks to this goal (normalised)**			**-0.577 (0.415)**
**Goal away team between start of minute 45 and half time**	**0.109 (0.229)**	**0.096 (0.229)**	**0.108 (0.229)**
Goal difference at half time	0.957*** (0.091)	0.974*** (0.094)	0.968*** (0.092)
Number of goals home team during first half	-0.011 (0.115)	-0.016 (0.116)	-0.025 (0.116)
Last goal in first half by home team	0.222 (0.142)	0.213 (0.142)	0.231 (0.142)
Last goal in first half by away team	0.015 (0.167)	0.038 (0.168)	0.036 (0.167)
Game in UEFA Europa League	-0.043 (0.147)	-0.047 (0.147)	-0.045 (0.147)
Game in group phase	-0.045 (0.102)	-0.049 (0.102)	-0.053 (0.102)
Red card in first half for home team	-0.887** (0.362)	-0.886** (0.362)	-0.860** (0.362)
Red card in first half for away team	-0.032 (0.233)	-0.035 (0.233)	-0.028 (0.233)
Relative strength home team	-0.023 (0.093)	-0.022 (0.093)	-0.030 (0.093)
Intercept	0.455 (0.351)	0.453 (0.351)	0.467 (0.351)
Fixed effects home team	Yes	Yes	Yes
Fixed effects away team	Yes	Yes	Yes
Dependent variable: Goal difference at full time	Yes	Yes	Yes
R^2^	0.671	0.671	0.671
N	1,179	1,179	1,179

Notes: The presented statistics are linear regression model estimates. The estimation results for the model’s main explanatory variables are in bold. A definition of the variables adopted in the regressions can be found in Section 2.1. The variables interacted with “Goal home team between start of minute 45 and half time” are reduced with their average among the observations for which “Goal home team between start of minute 45 and half time” equals 1. Standard errors are between parentheses.

*** (**) ((*)) indicate significance at the 1% (5%) ((10%)) significance level.

### 3.4 Robustness checks

In addition to the analyses presented in this article, we conducted many alternative analyses to check the robustness of our results. Firstly, for all analyses presented in this article, we also run the corresponding (ordered) logistic regressions. However, in the end we opted to present linear models, given (i) our overall focus on continuous dependent variables; (ii) the good performance of the linear probability model with White-corrected standard errors for binary dependent variables [53]; and (iii) the incidental parameters problem when combining logistic regression models with fixed effects [54]. Secondly, we re-estimated all regression models without excluding the games with no competitive value (see Section 2.1). Thirdly, the alternative soccer myth was tested for other definitions of “at the start of the second half” (first minute, first two minutes, first three minutes and first four minutes instead of first five minutes). Fourthly, various alternative specifications were tested with respect to our control variables, among which were (i) half time score dummies instead of the combination of two continuous variables used in our benchmark regression; (ii) season-specific team fixed effects instead of regular team fixed effects; and (iii) random effects at the home (away) team level instead of fixed effects at this level. Fifthly, various interactions other than those presented in Table 6, between scoring behaviour just before half time by the home team and game events and characteristics, were explored—for example, interactions with (i) non-linear alternatives for the relative strength of the teams; (ii) non-linear specifications for the goal difference at half time; (iii) a dummy for a non-negative (instead of positive) goal difference thanks to the goal just before half time; and (iv) a dummy for games in the group stage of the competitions. Finally, dimensions of heterogeneity in the overall zero effect of a goal just before half time by the away team (instead of the home team) were also tested. However, none of these analyses, the results of which are available on request, led to conclusions other than those presented in the former subsections.

## Discussion

In this section, we elaborate on potential explanations for our main finding of a non-positive effect of scoring just before half time on full time achievement. We structure this discussion following the clusters of success factors in professional soccer outcomes mentioned in Section 1: (i) team and coach ability; (ii) team effort; (iii) mental and physical condition of the team players; and (iv) choices made by these players, their coach and the referee. As team and coach ability is constant in the short term, no explanations for our findings related to this factor are put forward.

Firstly, a goal just before half time may lead to decompression—that is, a reduction of pressure. This might be the case especially for teams playing at their home ground in general and particularly when attendance (relative to the stadium’s capacity) is high [11, 55–56]. A goal just before half time by the home team can make the difference between being sent to the locker rooms with supportive applause or with pressurising boos. As a consequence, a (home) team that scores a goal just before half time may (unconsciously) relax a bit and go into the break with a feeling of having accomplished something. While believers of the soccer myth under investigation may perceive decompression as an asset, peer-reviewed literature shows that it can lead to lower performance [57–59]. Translated to the setting of a soccer game, the decompression related to a goal just before half time may lead to complacency rather than mobilisation of more effort during the second half. This explanation is supported by the observation that our main finding is related to the home team’s lower probability of scoring a goal during the second half (rather than due to a higher probability of scoring a goal during the second half by the away team) after a goal just before half time.

A second explanation goes the other way around. Players who leave the soccer pitch with a fresh goal may feel an elevation in ego, status, and position. Again, this may be the case especially for the home team, as supporters may support this egotism [55]. Peer-reviewed literature relates this rise in self-consciousness to more pressure to deliver (ergo, to cope with expectations) and, subsequently, to choking under pressure [16,48–50,60]. As a consequence, scoring just before half time may backfire on the mental status of the (home) team and thereby affect subsequent performance negatively.

Finally, the emotion of a goal just before half time may affect the tactical decisions trainers propose during the break [57,61]. If a goal just before half time obscures the assessment of the relative strength of the competing teams, this may yield tactical decisions which are not taken on a rational basis. Again, this problem might be more crucial for home teams. Home teams are, in general, expected to (and found to) follow a more offensive strategy than away teams [11,43]. It is often suggested that, while the away teams’ more defensive tactics are less complex, this more offensive strategy for home teams results in a constant struggle for a balance between scoring and playing attractive soccer on the one hand, and not letting the away team counterattack on the other [11,44]. The emotion of a goal just before half time might be a source of imbalance in this respect.

Our finding of a negative effect of scoring just before half time on full time achievement for home teams contrasts to some extent with the two studies finding no significant evidence for the absolute times of previous goals as a determinant of later game outcomes mentioned in Section 1. However, it is important to stress that these studies did not estimate this effect for home and away teams separately, as we did. In addition, they analysed goal scoring behaviour in other settings (national leagues and games with particular scores at half time only) than ours. We return to this point at the end of the next section.

## Conclusion

This study was, to the best of our knowledge, the first attempt in peer-reviewed literature to test the soccer myth that a particularly good moment to score a goal is just before half time. To this end, we constructed a unique data set containing rich information on 1,179 games played in the UEFA Champions League and the UEFA Europa League between 2008 and 2014. Our analysis of these data did not support the myth. In contrast, conditional on the goal difference and other game events and characteristics at half time, the final goal difference to the advantage of the home team turned out to be 0.520 goals lower in case of a goal just before half time by this team. We showed that this main finding relates to the home team’s lower probability of scoring a goal during the second half (rather than due to a higher probability of a goal being scored during the second half by the away team) after scoring a goal just before half time. On the other hand, we found a robustly neutral effect of scoring just before half time by the away team on full time achievement.

Besides the core finding that the soccer myth under investigation does not survive the confrontation with a first scientific evaluation (and, by extension, that trainers and players should not particularly chase a goal just before half time), our main results incorporate another relevant take-away message for the peer-reviewed literature on the determinants of achievement in soccer. Like Mechtel et al. [44], we concluded that the success factor under investigation in our study has a fundamentally heterogeneous effect depending on the home versus away status of the team. In the discussion of our results, we linked this finding to psychological dynamics which may essentially differ according to this status. As a consequence, we believe that further research on the determinants of soccer achievement must not neglect this natural dimension of heterogeneity.

We end this article by briefly highlighting three additional directions for further research related to limitations inherent to our study. Firstly, our results can be given a causal interpretation when one is willing to assume that, after controlling for the game events and characteristics included in our analyses, the event of scoring a goal just before half time (instead of at another moment) is random. We aimed to include all game events and characteristics which, based on our reading of the literature, could be expected to correlate with the event of a goal just before half time and full time achievement, but it is still possible that the association under investigation is confounded by other, unobserved events and characteristics. Therefore, although we do not see identification strategies which could (quasi) perfectly mimic random assignment of scoring behaviour just before half time, we are in favour of further research analysing the empirical value of the soccer myth central to this study by means of alternative statistical strategies. Secondly, while we were able to offer potential explanations supported by peer-reviewed literature for the non-positive association found between full time achievement and scoring just before half time, our analyses do not allow us to disentangle the exact empirical importance of these explanations. Therefore, we are in favour of future research, potentially including qualitative types of analysis, exploring the actual relevance of these mechanisms. This future research has the potential to yield insights relevant to more classical fields such as labour economics and labour psychology—for example, about timing of incentives and performance [62–63]. Thirdly, our results cannot be automatically generalised to other competitions where the home and away status of the teams is less outspoken, such as (i) FIFA World Cups, where there are no real home and away teams, and (ii) national leagues, where the distance in geographical and cultural terms is smaller [13,46,51]. Therefore, we are in favour of future research replicating our analyses based on data from other competitions.

## Supporting information

S1 Dataset (XLSX)
